# Factors associated with antenatal care utilization among women with disabilities in Ethiopia: *Using Andersen’s Behaviour Model of Health Service Use*

**DOI:** 10.1371/journal.pgph.0004759

**Published:** 2025-06-16

**Authors:** Abebe Alemu Anshebo, Yilma Markos, Sujit Behera, Natarajan Gopalan

**Affiliations:** 1 Department of Epidemiology and Public Health, School of Life Science, Central University of Tamil Nadu, Thiruvarur, Tamil Nadu, India; 2 Department of Midwifery, College of Medicine and Health Science, Wachemo University, Hosanna, Ethiopia; 3 Department of Public Health, College of Medicine and Health, Wachemo University, Hosanna, Ethiopia; PROPUL Evidence LLP, INDIA

## Abstract

Quality antenatal care is critical for reducing the risk of adverse pregnancy and childbirth outcomes such as abortion, stillbirth, preterm birth, and perinatal death. However, women with disabilities continue to face numerous barriers to accessing equitable and high-quality antenatal care services in low- and middle-income countries, including Ethiopia. Therefore, this study aimed to assess antenatal care utilization and associated factors among women with disabilities in the Central Ethiopia Regional State, Ethiopia. A community-based cross-sectional study was conducted, and a multistage random sampling technique was used to recruit 572 participants. The data were collected by face-to-face interviews using structured questionnaires. The Kobocollect mobile app was used to collect the data. A multivariate analysis evaluated the significant association between the explanatory variables and the utilisation of antenatal care services. The Andersen Behavioural Model of Health Service Use framework was used to categorise the predictors of antenatal care. Only 47.3% [95% CI: 43.1, 50.7] of pregnant women with disabilities used antenatal care [ANC]. Predisposing factors like higher women’s education, employment, awareness of ANC services, increasing gravidity and planned pregnancy; enabling factors like enrolling in community-based health insurance, availability of disability-inclusive health services, transportation system and health facility infrastructure; and need factor [experience of pregnancy-related complication during pregnancy] were found to have a statistically significant positive effect on ANC utilization among women with disabilities. The utilisation of antenatal care among pregnant women with disabilities was relatively low, which is attributed to predisposing, enabling and need factors. This finding underscores that the Ministry of Health and other stakeholders should pay more attention to enhancing awareness of ANC service and pregnancy planning, ensuring disabilities-sensitive healthcare services and disability-friendly health facilities and transportation to improve antenatal care services utilization by women with disabilities, which helps to meet Sustainable Development Goal 3.1, which aims to reduce maternal mortality to less than 70/100,000 live births by 2030.

## Introduction

According to the World Report on Disability, 15% of the global population experiences a disability within their lifetime, making them the world’s largest minority group. Approximately 80% of people with disabilities reside in low- and middle-income countries [LMICs] [1].

The Convention on the Rights of Persons with Disabilities [CRPD] affirms that women with disabilities have the right to receive equitable and high-quality maternal healthcare during the prenatal, delivery, and postnatal periods to achieve the best possible outcomes for mothers and babies [2]. The SDG 2030 Agenda prioritizes universal health coverage [UHC] as a critical component of overall health goals and places considerable emphasis on promoting access to safe, effective, and high-quality essential healthcare services at all levels [3,4].

The World Health Organization [WHO] defines antenatal care [ANC] as the care given to pregnant women by qualified healthcare professionals to ensure the best possible health conditions for the mother and the unborn child during pregnancy as well as recommends initiating antenatal care contact within the first 12 weeks of pregnancy and having at least eight contacts to prevent maternal and perinatal mortality [5]. High-quality antenatal care is critical for reducing the risk of adverse pregnancy and childbirth outcomes such as abortion, stillbirth, preterm birth, and perinatal death [6].

WHO declared that people living with disabilities deserve equal access to quality healthcare; however, women with disabilities have the highest rate of preventable maternal and perinatal mortality, as well as continue to have little or no access to essential, quality prenatal care services in resource-limited settings [7,8]. Unfortunately, despite substantial international efforts to promote and implement some initiatives to improve the accessibility of maternal healthcare services to women with disabilities, they continue to face significant barriers to accessing these services [9,10].

In low- and middle-income countries, studies have shown that women with disabilities encounter barriers to accessing maternal healthcare services, such as lack of family support, poor transportation, social unacceptance, and community discrimination [11–13]. Additionally, studies revealed that low income, poor education, unemployment, lack of information, erroneous assumptions, providers’ negative attitudes, violence, lack of insurance coverage, inaccessible and unavailability of service, disability-unfriendly physical structures, high costs, and distance to health facilities influence access to maternity care for women with disabilities [14–16].

In this study, Andersen’s behaviour model of health services use [BMHSU] is used identify factors associated with prenatal care service utilization among women with disabilities. The model helps to understand health services utilization and identify the factors that influence woman’s decision to use or not to use existing health services. The model categorizes the factors into three components; predisposing factors [e.g., demographic, social structure, and health beliefs characteristics], enabling factors [e.g., social resources, personal income, and health system resources], and need factors [e.g., perceived need and evaluated health status] influence a woman’s access to prenatal care services [17–19]. ANC utilization is the best strategy to reduce mortality in this era of high maternal morbidity and mortality through early detection, appropriate management, and timely referral of women with complications [20–22].

In Ethiopia, although several studies have examined the use of antenatal care services and associated factors among women without disabilities [23–26], little is known about antenatal care utilization and the challenges that women with disabilities encounter while seeking prenatal care services in low-income countries, including Ethiopia. Thus, this study aimed to assess antenatal care service utilization and associated factors among pregnant women with disabilities in the Central Ethiopia Regional State, Ethiopia.

## Methods

### Study design, period, setting, and study population

A community-based cross-sectional study was used from December 2023 to February 2024 in the Central Ethiopia Regional State, Ethiopia. In the region, there are seven zones and three special districts. The region comprises Guragem Hadiya, Silt’e, Halaba, Kambata, and Yem zones, as well as Kebena, Mareko, and Tembaro special woredas’. According to the National Statistical Agency population projection report [2023/24], the total population is estimated at around 6,430,235; of these people, 3,243,411 [50.44%] are female and 3,186,824 [49.56%] are male. Approximately 100,000 people are living with disabilities, with females accounting for 50% of this population [27]. All pregnant women with disabilities in the study area were considered the source population, while the selected pregnant women with disabilities were considered the study population.

### Sample size determination

The sample size was determined using a single population proportion formula considering the following assumptions: a previous study proportion of 65.6%, a 95% confidence interval [60.4, 70.6] [28], a critical value of the 95% confidence level [z = 1.96]*,* a margin of error of 5% a design effect of d = 1.5, and considering a nonresponse of 10%. The final sample included 572 participants.

### Sampling procedure

This study employed a multistage random sampling technique to recruit study participants. Initially, three zones were selected randomly from the Central Regional State of Ethiopia. Secondly, from each zone, four districts were selected at random; thirdly, from each district, six kebeles or clusters were selected at random. Finally, Seventy-two kelebes were considered in this study. The smallest government administrative unit in Ethiopia is a kebele, which represents the entire population. The sample size was proportionally allocated to each kebele. The list of all pregnant women with disabilities in the selected clusters was obtained from the registration book held by health extension workers in each cluster/Kebele. Finally, simple random sampling techniques were used to enroll study participants.

### Inclusion and exclusion criteria

Pregnant women with visual, hearing, speech, or physical impairment who were permanent residents [who lived more than six months] in the selected cluster at the time of data collection were included. However, the study did not include seriously ill women or women with cognitive impairments. Most importantly, excluding women with cognitive impairment was needed because such impartments could hinder the women’s ability to understand and respond to the study questionnaires or recall past events accurately, potentially compromising data quality.

### Data collection

Face-to-face interviews with pretested, structured questionnaires were used to collect data. Data was collected by Health Extensions Works, along with body language expertise. The questionnaires were designed in English and translated into the local language by experts. Predisposing, enabling, and need factors, as well as antenatal care service utilisation by women with disabilities, were covered by the questionnaire. The data collection tool was developed from previous studies and surveys and contextualised based on Andersen’s behavioural model [17,29–32]. The Kobocollect mobile data collection tool was used to collect and clean the data, and then exported to the Statistical Package for Social Sciences [SPSS] version 26 for analysis.

### Data analysis

Data was exported in XLS form from the KoboTool box to the Statistical Package for Social Science [SPSS] 26 version for analysis. Descriptive results were reported by frequencies and proportions in the tables and figures. Bivariate and multivariate analyses were used to evaluate the associations between explanatory variables and the outcome variables. Multicollinearity is used to measure the correlation between predictor variables. The Hosmer-Lemeshow test was used to check model fitness. The multivariable logistic regression model included all the significant variables in the bivariate analysis [p < 0.25]. The strength of the associated factors and outcome variables was expressed as an adjusted odds ratio [AOR] with a 95% confidence interval [CI]. At p < 0.05, the association of variables was found to be statistically significant.

### Outcome variable

The WHO defines antenatal care [ANC] as the care given to pregnant women by skilled healthcare professionals to ensure the best possible health outcomes for both the mother and the fetus throughout pregnancy [5]. However, in this study, antenatal care utilization was defined as those pregnant women with disabilities who had at least one ANC contact with a skilled healthcare professional during pregnancy at the time of data collection.

### Explanatory variables

According to Andersen’s Health Services utilization model, predisposing factors [demographic and reproductive characteristics], enabling factors [resources, health facilities, transportation system, community-based health insurance, and reproductive knowledge], and need factors [pregnancy-related complications] were considered explanatory variables [17,32].

### Ethical approval and consent to participate

The study obtained ethical approval from the Research Ethical Committee of Wachemo University and the Research Advisory Committee of the Central University of Tamil Nadu, as referred to in Ref. No. WCU-IRB 0021/23. The study participants were informed about the study’s purpose and potential risks and benefits. Informed written consent was obtained by their signature or a thumbprint from each study participant, and confidentiality was ensured by keeping the data anonymous.

## Results

### Sociodemographic characteristics of study participants

Out of 572 participants, 562 responded to the questionnaire thoroughly, yielding a response rate of 98.2 per cent. The mean age of the study participants was 30 [±3.6] years. The majority of study participants [80.15%] were married. Nearly half [50.4%] of the study participants could not read or write, and 374 [66.5%] were not employed. Regarding impairment status, physical or extremity impairment accounts for 62.5 per cent of study participants, as presented in Table 1.

**Table 1:** Sociodemographic characteristics of pregnant women with disabilities in the Central Ethiopia Regional State, Ethiopia, 2024 [N = 562].

Variable	Categories	Frequency	Percentage
Age	<= 24	61	10.9
	25-34	381	67.8
	>= 35	120	21.4
Marital status	Married	450	80.1
	Single	63	11.2
	Others ^a^	49	8.7
Women’s education level	No formal education	283	50.4
	Formal education	279	49.6
Husband’s education level	No formal education	100	22.2
	Formal education	350	77.8
Residence	Rural	459	81.7
	Urban	103	18.3
Religion affiliation	Protestant	374	66.5
	Orthodox	110	19.6
	Muslim	75	13.3
	Other ^b^	3	0.5
Women’s Employment	Not employed	374	66.5
	Employed	188	33.5
Husband’s Employment	Not employed	141	31.3
	Employed	309	68.7
Household size	≤5	486	86.5
	> 5	76	13.5
Disability types	Hearing	57	10.1
	Vision	134	23.8
	Speech	19	3.4
	Extremities/physical	352	62.6

^a^Divorced, Widowed/separated

^b^Catholic, traditional religion.

### Reproductive health-related characteristics of the study participants

Most study participants [59.1%] were multigravida, and three hundred sixteen [56.2%] planned pregnancy. Two hundred ninety-nine [53.2%] of study participants had heard about antenatal care, whereas two hundred sixty-three [46.8%] had not. Regarding antenatal care booking, 54.9% in the first, 38.0% in the second, and 7.1% in the third trimester initiated ANC contact, respectively. One hundred forty-nine [26.5%] commenced antenatal care contact due to health issues (Table 2).

**Table 2:** Reproductive health-related characteristics of pregnant women with disabilities in the Central Ethiopia Regional State, Ethiopia [N = 562].

Variables	Categories	Frequency	Percentage
Gravidity	Primigravida	230	40.9
	Multigravida	332	59.1
Planned pregnancy	Yes	316	56.2
	No	246	43.8
History of abortion	Yes	88	26.5
	No	244	73.5
Pregnancy-related complications during pregnancy	Yes	95	16.9
	No	467	83.1
Awareness of ANC	Yes	299	53.2
	No	263	46.8
Source of information	Health workers	265	47.2
	Radio/TV	14	2.5
	Social media	2	0.4
	Neighbours/relatives	18	3.2
Know the time to initiate ANC contact	Yes	140	24.9
	No	159	28.3
Know the number of ANC contacts	Yes	77	13.7
	No	222	39.5
Reason to initiate ANC contact	Regular check-up	117	20.8
	Health problem	149	26.5
ANC contact was initiated in	1^st^-trimester	146	54.9
	2^nd^-trimester	101	38.0
	3^rd^-trimester	19	7.1

### Antenatal care service utilisation-related characteristics

As presented in Table 3, 347 [61.7%] participants responded that ANC services were inequitable and insensitive to women with special needs, while 215 [38.3%] responded that the services were equitable and sensitive. Four hundred fifteen [73.8%] reported that the transportation system was inaccessible to women with disabilities, 391 [69.6%] experienced unfriendly health infrastructure, 402 [71.5%] reported discrimination in health service delivery, and 450 [80.1%] weren’t supported by the community to use antenatal care services.

**Table 3:** Antenatal care utilisation-related characteristics of pregnant women with disabilities in the Central Ethiopia Regional State, Ethiopia [N = 562].

Variables	Categories	Frequency	Percentage
Equitable and sensitive antenatal care	Yes	215	38.3
	No	347	61.7
Disability-inclusive transportation system	Yes	147	26.6
	No	415	73.8
Time spent to reach health facilities	≤ 1 hour	65	15.7
	> 1 hour	350	84.3
Disability-inclusive health facilities	Yes	171	30.4
	No	391	69.6
Discrimination in service delivery	Yes	402	71.5
	No	160	28.5
Unsupportive societal/community perception of ANC use	Yes	450	80.1
	No	112	19.9
Spousal role in ANC initiation	Yes	295	52.5
	No	267	47.5
Enrolled in the community-based health insurance [CBHI] scheme	Yes	162	28.8
	No	400	71.2
Preventive measures at ANC contact^*^	Yes	187	33.3
	No	79	14.1
Nutritional measures at ANC contact^*^	Yes	196	34.9
	No	70	12.5
Maternal condition assessment at ANC contact^*^	Yes	245	43.6
	No	21	3.7
Fetal condition assessment at ANC contact^*^	Yes	175	31.1
	No	91	16.2
ANC contact initiated^*^	1^st^-trimester	146	54.9
	2^nd^-trimester	101	38.0
	3^rd^-trimester	19	7.1

* Indicates the denominator is 266; the women who initiated ANC contact only.

Regarding services that pregnant women with disabilities were given during ANC contact, 187 [33.3%] received preventive measures [screening for infection, vaccination and lifestyle advice], 196 [34.9%] received nutritional measures [Iron and folic acid supplementation, having a balanced diet and increased calorie intake], 245[43.6] received maternal condition assessments [weight and blood pressure, urine analysis and checking for anaemia and physical examination], and 175 [31.1%] received fetal condition assessments [Fetal heart rate measure and ultrasound]. Among women who initiated ANC contact, one hundred forty-six [54.9%] women booked antenatal care contact in the first trimester [week 1–12 of gestation] as per the WHO guideline.

### The prevalence of antenatal care utilisation among women with disabilities

**Fig 1:**
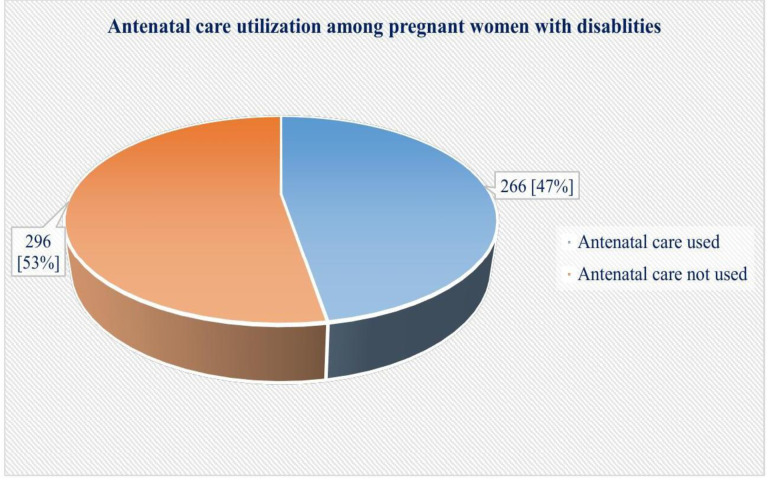
The magnitude of antenatal care utilisation among women with disabilities in the Central Ethiopia Regional State, Ethiopia [N = 562].

In this study, 266 [47.3%] of 562 participants used ANC services, whereas 296 [52.7%] did not use ANC services at the time of data collection as per the WHO guide recommendation (Fig 1).

**Fig 2:**
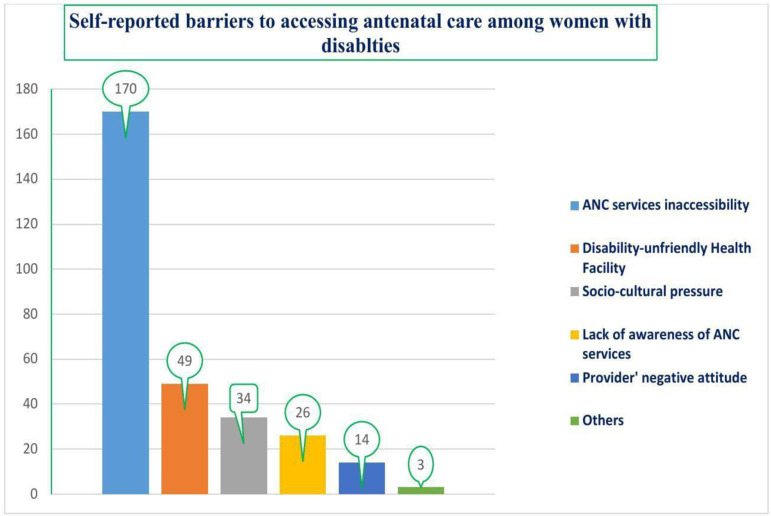
Barriers to accessing antenatal care among women with disabilities in the Central Ethiopia Regional State, Ethiopia [n = 296].

According to the participants’ self-report, the barriers to using ANC services included lack of awareness of ANC services, inaccessibility of the ANC services, disability-unfriendly health facility infrastructure, providers’ negative attitude, sociocultural beliefs, and others [delay in deciding to seek ANC services] (Fig 2).

### Factors associated with antenatal care utilisation among pregnant women with disabilities

Antenatal care is an important part of comprehensive maternal health care because it allows for timely interventions and provides women with the knowledge and awareness they require for a safe pregnancy and childbirth. However, this study found that the prevalence of antenatal care [ANC] service utilisation was relatively low [47.3%] among pregnant women with disabilities [PWWD] and significantly associated with some predisposing, enabling and need factors.

To identify significantly associated factors with antenatal care utilisation, the multivariable logistic regression included all candidate variables with a p-value less than 0.25 in the binary analysis. After controlling the confounders in the multivariable logistic regression, women’s education level [literate, AOR = 1.50, 95% CI: 1.21, 2.32, p = 0.007], women’s employment [employed, AOR = 3.03, 95% CI: 1.85, 4.95, p < 0.0001], primigravida [AOR = 0.15, 95% CI: 0.09, 0.24, p < 0.0001], unplanned pregnancy [AOR = 0.39, 95% CI: 0.25, 0.63, p < 0.0001], experience of pregnancy-related complication [AOR = 2.72, 95% CI: 1.61, 4.59, p < 0.0001], women’s awareness of antenatal care [AOR = 0.058, 95% CI: 0.05, 0.15, p < 0.0001], CBHI scheme [AOR = 2.38, 95% CI: 1.47, 3.86, p < 0.0001], disability-sensitive service [AOR = 2.30, 95% CI: 1.33, 3.97, p = 0.003], transportation system [AOR = 2.06, 95% CI: 1.07, 3.93, p = 0.029], and disability-unfriendly health facilities structure [AOR = 2.56, 95% CI:1.45, 4.52, p = 0.001] were significantly associated with ANC services utilization among pregnant women with disabilities as presented in Table 4.

**Table 4:** Significantly associated factors with antenatal care utilisation among women with disabilities in the Central Ethiopia Regional State, Ethiopia [N = 562].

Variable	ANC services use		95% CI for Exp. [B]		p-value
	No [N, %]	Yes [N, %]	COR	AOR	
Women’s education level					
Illiterate	172 [58.1]	111 [41.7]	1.00		
Literate	124 [41.9]	155 [58.3]	1.94 [1.38, 2.71]^*^	1.5 [1.21, 2.32]^**^	0.007
Women’s Employment					
Not employed	222 [75.0]	152 [57.1]	1.00	1.00	
Employed	74 [25.0]	114 [42.9]	2.25 [1.57, 3.22]^*^	3.03 [1.85,4.95]^**^	0.0001
Gravidity [number of pregnancies]					
Primigravida	175 [59.1]	55 [20.7]	0.18 [0.12, 0.26]^*^	0.2 [0.09, 0.24]^**^	0.0001
Multigravida	121 [40.9]	211 [79.3]	1.00	1.00	
Pregnancy planned					
Yes	144 [48.6]	172 [64.7]	1.00	1.00	
No	152 [51.4]	94 [35.3]	0.52 [0.37, 0.73]^*^	0.39 [0.2, 0.63]^**^	0.0001
Experience pregnancy-related complications					
Yes	32 [10.8]	63 [23.7]	2.6 [1.61, 4.07]^*^	2.7 [1.6, 4.59]^**^	0.0001
No	264 [89.2]	203 [76.3]	1.00	1.00	
Enrolled in the CBHI program					
Yes	47 [15.9]	115 [43.2]	4.03 [2.72, 5.99]^*^	2.38 [1.5, 3.86]^**^	0.0001
No	249 [84.1]	151 [56.8]	1.00	1.00	
ANC services awareness					
Yes	71 [24.0]	228 [85.7]	1.00	1.00	
No	225 [76.0]	38 [14.3]	0.50 [0.30, 0.80]^*^	0.48 [0.35, 0.65]^**^	0.0001
Disability-sensitive ANC service					
Yes	66 [22.3]	149 [56.0]	4.4 [308, 6.39]^*^	2.30 [1.3, 3.97]^**^	0.003
No	230 [77.7]	117 [44.0]	1.00	1.00	
Disability-inclusive transportation system					
Yes	30 [10.1]	117 [44.0]	6.96 [4.5, 10.90]^*^	2.06 [1.1, 3.9]^**^	0.029
No	266 [89.9]	149 [56.0]	1.00	1.00	
Disability-inclusive health facilities infrastructure					
Yes	46 [15.5]	125 [47.0]	4.82 [3.24, 7.16]	2.6 [1.45, 4.52]^**^	0.001
No	250 [84.5]	141 [53.0]	1.00	1.00	

& widowed, divorced

* P < 0.25

** P < 0.05 [indicates statistical significance].

## Discussion

High-quality antenatal care service is essential for individuals, society, and future generations since it contributes to the reduction of preventable maternal and perinatal morbidity and mortality. This study aimed to assess the prevalence of antenatal care utilization and associated factors among pregnant women with disabilities in the Central Ethiopia Region State, Ethiopia.

From the Perspective of Andersen’s Behaviour Model, this study revealed that predisposing factors [such as women’s education, employment, gravidity, awareness of antenatal care, and pregnancy planning], enabling factors [including enrolling in community-based health insurance, disability-sensitive services, disability-inclusive transportation systems, and disability-inclusive health facilities], and need factors [pregnancy-related complications] were significantly associated with antenatal care service utilization among pregnant women with disabilities in the Central Ethiopia Regional State, Ethiopia.

This study found that only 47.3% [95% CI: 43.1, 50.7] of pregnant women with disabilities used antenatal care services. This finding is lower than 62% from EDHS 2016 report [33], the 74% from the Ethiopian Mini-Demographic Health Survey finding [2019] [34], the 63.77% from a systematic review and meta-analysis [24], the 96% in Cape Town, South Africa, reported by Gichane et al. [2017] [35], and the 55.66% and 58.53% findings from the multi-country analysis reported by Chilot et al. [2023] and Tessema et al. [2021], respectively [36,37]. The possible explanation for inconsistent results might be differences in demographics [such as education, employment, and residence], socio-economic backgrounds, cultural context of study populations, methodological approach, measurement of outcome variable, study settings, and some context-specific factors like healthcare access.

Women’s education status and employment are significantly associated with antenatal care [ANC] use among pregnant women with disabilities. Literate womens’ were 1.5 times more likely than non-literate [cannot read and write] women to use antenatal care services. Similarly, employed women were three times more likely to utilize antenatal care services than their counterparts. This study result is supported by studies conducted in Ethiopia by Kidie et al. [2024] [38], Ayalew et al. [2022], and Tekelab et al. [2019] [24,39]. One explanation could be that education and employment are critical determinants factors that enhance understanding of the importance of antenatal care and enable women to make informed choices and initiate timely antenatal services, even in the face of stigma or societal challenges related to disabilities. Furthermore, women’s employment improves their economic status in the community, access to health insurance, and decision-making power on health matters.

Our findings indicate that the number of pregnancies or gravidity and the utilization of antenatal care had a significant association. The result indicated that the primigravida women were 80 per cent less likely to use antenatal care services than the multigravida women. These findings are consistent with research conducted in Uganda by Natukunda B et al. [2022] [40]. On the other hand, studies conducted in Somaliland and Bangladesh showed that primigravida women received more antenatal care services compared to multigravida [41,42]. The possible reasons for the inconsistency of the result might be variations in the study population [such as demographic differences and cultural contexts], study design, access to healthcare services [availability, accessibility and quality of service], awareness and knowledge level and health system factors.

This study found that pregnancy planning was significantly associated with antenatal care. Those women who became pregnant unintentionally were 61 per cent less likely to use antenatal care services as compared to women who had planned pregnancies. This finding aligns with previous studies that consistently show that planned pregnancy positively influences antenatal care use, as reported by Demissie et al. [2024] [43] and Tesfaye et al. [2017] in Ethiopia [44] and by Ahinkorah et al. [2021] in Guinea [45]. One possible reason for this association is that women who had unplanned pregnancies may be less likely to seek antenatal care services because of the lack of psychological readiness to take responsibility. Moreover, unplanned pregnancies lead to delays in recognising the need for antenatal care and a lack of partner support, which leads to emotional distress and makes them less likely to initiate ANC.

Another significant factor associated with the use of ANC services among women with disabilities was the presence of pregnancy-related complications. Women who experienced pregnancy-related complications during pregnancy were 2.7 times more likely to receive antenatal care services when compared to their counterparts. This finding is consistent with research conducted in Ethiopia by Tolera et al. [2020], which found that pregnant women with a high fever were seven times more likely to use antenatal care services [46]. The possible explanations might be complications such as discomfort, anxiety, severe nausea, hypertension, and bleeding, which often trigger the health-seeking behaviour and motivate pregnant women to seek ANC contacts, receive good encouragement from families or partners to decide timely and visit health facilities to get essential ANC services. This relationship underscores that further health education programs or campaigns are needed to improve awareness of women and families about the importance of ANC use and to make it a routine preventive care, not only for complications.

A study found that there was a significant association between antenatal care service utilisation and women’s awareness towards antenatal care services. The result showed that women who did not have information about ANC were 52 per cent less likely to utilise antenatal services than their counterparts. This result aligns with previous study findings reported by Tesfaye et al. [2017] and Alemayehu et al. [2020] in Ethiopia [44,47], Haddrill et al. [2014] [48], and Aziz Ali et al. [2020] in Pakistan [49]. One possible explanation is that women are more likely to seek maternal healthcare services if they have information and understanding about the ANC services from different sources. This underscores that awareness and knowledge about the ANC services are key to ensuring ANC service utilization among pregnant women with disabilities.

The current study found that antenatal care services utilisation was significantly associated with community-based health insurance [CBHI] enrollment. Women enrolled in the CBHI program had a twofold higher likelihood of using antenatal care services than not enrolled in CBHI. This result is consistent with the previous study findings reported by Tang et al. [2019] in China [50]. The possible reasons for this association could be that CHB insurance could reduce out-of-pocket healthcare expenses and improve motivation to utilise ANC services because the financial burden is low, and enrollment improves awareness through community-level sensitisation programs. These findings show that CBHI enrollment improves health literacy, and all stakeholders must endeavour to include all women with disabilities in the CBHI scheme.

Consistent with the previous studies findings [13,51,52], this study found that disability-sensitive ANC service was significantly associated with ANC service utilization, emphasizing the important role of inclusive healthcare practice in improving accessibility and availability of service for women with disabilities and antenatal care service utilization was twice more likely used among women who reported that the services were equitable and disability-sensitive. One possible explanation could be that when ANC services are disability-sensitive, women may not face attitudinal, communication and physical barriers, which help to access care easily, to address the unique needs of women, reduce stigma and discrimination, feel more comfortable, and improve their likelihood of using ANC services. This finding emphasises that ensuring disability-sensitive healthcare practices and programs has paramount significance in improving equitable access and enhancing health outcomes among pregnant women with disabilities [53].

The findings of this study revealed that antenatal care utilization among pregnant women with disabilities was significantly associated with disability-inclusive health facilities and transportation systems. In line with previous studies, this study’s findings showed that women who reported that the health facility structure was disability-friendly were almost threefold more likely to use antenatal care services, as well as those who reported that the transportation system was inclusive were twofold more likely to uptake antenatal care service utilization than their counterparts, which was constant with the previous study findings reported by Ganle et al. [2016] in Ghana [54], Natukunda B et al. [2022] in Uganda [40], Devkota et al. [2018] in Nepal [55], and A. Wolowicz et al. [2020] in Poland [56]. A possible explanation might be that when health facilities are physically and culturally accommodating, meaning [such as ramps, assistive technologies and trained personnel] and disability-inclusive transportation systems [including wheelchair-accessible vehicles and affordable transportation], women with disabilities are more likely to use antenatal care services. The result highlights the significance of ensuring an enabling environment to address the unique needs of women with disabilities, which aligns with the social model of disability perspective [57].

### Limitations of the study

This study has certain limitations. Since data was captured at a single point in time, the study cannot confirm a causal relationship and measure the adequacy or optimal use of the ANC services. Also, women with cognitive impairments were excluded, which may limit the generalizability of the result and may lead to an underestimation of the barriers faced by the most vulnerable groups. Moreover, data collectors were health extension workers; thus, social desirability bias might compromise the findings. Future longitudinal or qualitative studies are recommended to explore the lived experiences and challenges of women with different disabilities in the study area.

### Implications and scope of future research

The study results reveal a substantial disparity in accessibility of antenatal care services for women with disabilities. The healthcare system should develop tailored interventions, practices, and initiatives to ensure accessible and disability-inclusive antenatal care services for women with disabilities. Future research, longitudinal studies are suggested to explore a cause-and-effect relationship between Variables and lived experience or challenges of women while seeking antenatal care among women with disabilities in the study area. Additionally, specific disability focused researchers are suggested because different disabilities have disability specific challenges and require specific scientific suggestions and recommendations.

## Conclusion

In conclusion, the utilisation of antenatal care among pregnant women with disabilities was relatively low, which is attributed to some predisposing, enabling and need factors. This finding underscores the need for more attention from the Ministry of Health and other stakeholders to improve antenatal care accessibility for women with disabilities. Enhancing awareness of antenatal services and pregnancy planning, ensuring accessible and disability-sensitive services, and designing disability-inclusive health facilities and transportation are suggested strategies to improve the utilisation of antenatal care services by women with disabilities. Furthermore, these interventions are thought to contribute significantly to meeting Sustainable Development Goal 3.1, which aims to reduce maternal mortality to less than 70/100,000 live births by 2030.

## References

[1] The Lancet Global Health. Disability: measurement matters. Lancet Glob Health. 2021;9(8):e1028. doi: 10.1016/S2214-109X(21)00312-0 34297946

[2] Lawson A. United Nations Convention on the Rights of Persons with Disabilities [CRPD]. Int Eur Labour Law. 2018:455–61.

[3] World Health Organization. World health statistics - monitoring health for the SDGs. World Heal Organ. 2016;1.121.

[4] Krehbiel JN, Gabel MJ, Carrubba CJ. The European court of justice. Routledge Handb Judic Behav. 2017;16301:467–90.

[5] Hoffman DW. WHO recommendations on antenatal care for a positive pregnancy experience. 2016;100:102. Available from: http://www.who.int/about/licensing/copyright_form 28079998

[6] Tunçalp Ӧ, Were WM, MacLennan C, Oladapo OT, Gülmezoglu AM, Bahl R, et al. Quality of care for pregnant women and newborns-the WHO vision. BJOG. 2015;122(8):1045–9. doi: 10.1111/1471-0528.13451 25929823 PMC5029576

[7] Carroll A. World report on disability. Ir Med J. 2012;105(5):3–7.

[8] Caliskan Y. The global strategy for women’s, children’s and adolescents’ health [2016-2030]. UN Secr. 2016;31–48. 10.2471/BLT.16.174714 PMC4850547 27147756

[9] Barbera JP, Cichon B, Ankam N, Schwartz BI. Equitable care for patients with disabilities: considerations for the gynecologic health care professional. Obstet Gynecol. 2024;143(4):475–83. doi: 10.1097/AOG.0000000000005493 38176014 PMC10953678

[10] Gréaux M, Moro MF, Kamenov K, Russell AM, Barrett D, Cieza A. Health equity for persons with disabilities: a global scoping review on barriers and interventions in healthcare services. Int J Equity Health. 2023;22(1):236. doi: 10.1186/s12939-023-02035-w 37957602 PMC10644565

[11] Nguyen TV, King J, Edwards N, Pham CT, Dunne M. Maternal healthcare experiences of and challenges for women with physical disabilities in low and middle-income countries: a review of qualitative evidence. Sex Disabil. 2019;37(2):175–201. doi: 10.1007/s11195-019-09564-9

[12] Black K, Kollodge R, Rabie D, Ratcliffe L, Trautwein C, Zerzan R, et al. UNFPA state of world population report - INTERWOVEN LIVES, THREADS OF HOPE. 2024. Available from: https://www.unfpa.org/sites/default/files/pub-pdf/swp2024-english-240327-web.pdf

[13] Blair A, Cao J, Wilson A, Homer C. Access to, and experiences of, maternity care for women with physical disabilities: A scoping review. Midwifery. 2022;107:103273. doi: 10.1016/j.midw.2022.103273 35158123

[14] Matin BK, Williamson HJ, Karyani AK, Rezaei S, Soofi M, Soltani S. Barriers in access to healthcare for women with disabilities: a systematic review in qualitative studies. BMC Womens Health. 2021;21(1):44. doi: 10.1186/s12905-021-01189-5 33516225 PMC7847569

[15] Ahumuza SE, Matovu JKB, Ddamulira JB, Muhanguzi FK. Challenges in accessing sexual and reproductive health services by people with physical disabilities in Kampala, Uganda. Reprod Health. 2014;11:59. doi: 10.1186/1742-4755-11-59 25086444 PMC4131806

[16] Eide AH, Mannan H, Khogali M, van Rooy G, Swartz L, Munthali A, et al. Perceived barriers for accessing health services among individuals with disability in four African countries. PLoS One. 2015;10(5):e0125915. doi: 10.1371/journal.pone.0125915 25993307 PMC4489521

[17] Andersen RM. Revisiting the behavioral model and access to medical care: does it matter? J Health Soc Behav. 1995;36(1):1–10. doi: 10.2307/2137284 7738325

[18] Kabir MR. Adopting Andersen’s behavior model to identify factors influencing maternal healthcare service utilization in Bangladesh. PLoS One. 2021;16(11):e0260502. doi: 10.1371/journal.pone.0260502 34843566 PMC8629289

[19] You H, Yu T, Gu H, Kou Y, Xu XP, Li XL, et al. Factors associated with prescribed antenatal care utilization: a cross-sectional study in Eastern Rural China. Inq. 2019;56. 10.1177/0046958019865435 PMC6681245 31370723

[20] Carroli G, Rooney C, Villar J. How effective is antenatal care in preventing maternal mortality and serious morbidity? An overview of the evidence. Paediatr Perinat Epidemiol. 2001;15(Suppl 1):1–42. 10.1046/j.1365-3016.2001.0150s1001.x 11243499

[21] Girotra S, Malik M, Roy S, Basu S. Utilization and determinants of adequate quality antenatal care services in India: evidence from the National Family Health Survey (NFHS-5) (2019-21). BMC Pregnancy Childbirth. 2023;23(1):800. doi: 10.1186/s12884-023-06117-z 37978458 PMC10657001

[22] Mwenebanda E, Machado A, Patel AI, Nyondo-Mipando AL, Chiumia IK. Factors influencing antenatal care attendance in the eight contact era policy: a case of selected maternal health service facilities in Blantyre, Malawi. BMC Pregnancy Childbirth. 2024;24(1):704. doi: 10.1186/s12884-024-06895-0 39462321 PMC11514860

[23] Tsegaye S, Yibeltal K, Zelealem H, Worku W, Demissie M, Worku A, et al. The unfinished agenda and inequality gaps in antenatal care coverage in Ethiopia. BMC Pregnancy Childbirth. 2022;22(1):82. doi: 10.1186/s12884-021-04326-y 35093008 PMC8801127

[24] Tekelab T, Chojenta C, Smith R, Loxton D. Factors affecting utilization of antenatal care in Ethiopia: A systematic review and meta-analysis. PLoS One. 2019;14(4):e0214848. doi: 10.1371/journal.pone.0214848 30973889 PMC6459485

[25] Birhanu F, Mideksa G, Yitbarek K. Are Ethiopian women getting the recommended maternal health services? The analysis of Ethiopian mini Demographic and Health Survey 2019. Health Sci Rep. 2022;5(6):e879. doi: 10.1002/hsr2.879 36248354 PMC9552992

[26] Wake SK, Botore A, Mohammed A, Gemede K, Bariso M, Gerema U. Disparities in Antenatal Care Visits between Urban and Rural Ethiopian Women. J Pregnancy. 2023;2023:9031344. doi: 10.1155/2023/9031344 37799709 PMC10550413

[27] Central Statistical Agency. Population projection of Ethiopia for all regions at Wereda Level from 2014 – 2017. J Ethnobiol Ethnomed [Internet]. 2013;3(1):28. Available from: http://www.csa.gov.et/images/general/news/pop_pro_wer_2014-2017_final

[28] Tenaw Z, Gari T, Gebretsadik A. Unintended pregnancy and its associated factors among women with disabilities in central Sidama National Regional State, Ethiopia: a multilevel analysis. BMC Pregnancy Childbirth. 2023;23(1):522. doi: 10.1186/s12884-023-05848-3 37460959 PMC10353093

[29] Ethiopia CSA. LSMS-Integrated Surveys on Agriculture Ethiopia Socioeconomic Survey [ESS]. Report. 2015;7–37.

[30] Barrett G, Smith SC, Wellings K. Conceptualisation, development, and evaluation of a measure of unplanned pregnancy. J Epidemiol Community Health. 2004;58(5):426–33. doi: 10.1136/jech.2003.014787 15082745 PMC1732751

[31] ICF CSA [CSA] [Ethiopia]. Ethiopia Demographic and Health Survey 2016: Key Indicators Report. 2016.

[32] Andersen R; Newman J. Societal and individual determinants of medical care utilization in the United States Author [s]: Ronald Andersen and John F. Newman Source: The Milbank Memorial Fund Quarterly Health and Society, Vol. 51, No. 1 [Winter, Published by: Wiley o. Milbank Mem Fund Q. 1973;51(1):95–124. 4198894

[33] CSA. Central Statistical Agency [CSA] [Ethiopia] and ICF [Internet]. Addis Ababa, Ethiopia, and Rockvile, Maryland, USA: Ethiopia Demographic and Health Survey; 2016. p. 134. Available from: www.DHSprogram.com

[34] Ethiopian Public Health Institute Addis Ababa. Ethiopia Mini Demographic and Health Survey. Ethiopia: Federal Democratic Republic of Ethiopia; 2019.

[35] Gichane MW, Heap M, Fontes M, London L. “They must understand we are people”: Pregnancy and maternity service use among signing Deaf women in Cape Town. Disabil Health J. 2017;10(3):434–9. doi: 10.1016/j.dhjo.2017.03.016 28416204 PMC5484522

[36] Chilot D, Belay DG, Ferede TA, Shitu K, Asratie MH, Ambachew S, et al. Pooled prevalence and determinants of antenatal care visits in countries with high maternal mortality: A multi-country analysis. Front Public Health. 2023;11:1035759. doi: 10.3389/fpubh.2023.1035759 36794067 PMC9923119

[37] Tessema ZT, Teshale AB, Tesema GA, Tamirat KS. Determinants of completing recommended antenatal care utilization in sub-Saharan from 2006 to 2018: evidence from 36 countries using Demographic and Health Surveys. BMC Pregnancy Childbirth. 2021;21(1):192. doi: 10.1186/s12884-021-03669-w 33676440 PMC7937261

[38] Kidie AA, Asmamaw DB, Belachew TB, Fetene SM, Baykeda TA, Endawkie A, et al. Socioeconomic inequality in timing of ANC visit among pregnant women in Ethiopia, 2019. Front Public Heal. 2024;12. 10.3389/fpubh.2024.1243433 PMC10972848 38550321

[39] Ayalew HG, Asefa KT, Liyew AM. Determinants of recommended antenatal care visits among pregnant women in Ethiopia: a generalized linear mixed-effects modeling. BMC Pregnancy Childbirth. 2022;22(1):867. doi: 10.1186/s12884-022-05213-w 36419025 PMC9685851

[40] Natukunda B, Musoke D, Kiconco A, Mugambe S, Atuhairwe C, Taremwa IM, et al. Maternal health-seeking behaviour of peri-urban women living with disability in Busiro South Health sub District, Wakiso district, Uganda: a community-based study. Afr Health Sci. 2022;22(4):396–407. doi: 10.4314/ahs.v22i4.45 37092094 PMC10117492

[41] Mouhoumed HM, Mehmet N. Utilization pattern of antenatal care and determining factors among reproductive-age women in Borama, Somaliland. J Prev Med Hyg. 2021;62(2):E439–46. doi: 10.15167/2421-4248/jpmh2021.62.2.1882 34604585 PMC8451356

[42] Islam MdA, Sathi NJ, Abdullah HM, Naime J, Butt ZA. Factors affecting the utilization of antenatal care services during pregnancy in Bangladesh and 28 other low- and middle-income countries: a meta-analysis of demographic and health survey data. Dr Sulaiman Al Habib Med J. 2022;4(1):19–31. doi: 10.1007/s44229-022-00001-2

[43] Demissie KA, Jejaw M, Wondimu BG, Mersha YT, Demsash ES, Dessie SG, et al. Only 9% of mothers have eight and more ANC visit in 14 sub-saharan African countries; evidence from the most recent DHS 2018-2023: a multilevel analysis. BMC Public Health. 2024;24(1):1631. doi: 10.1186/s12889-024-19145-x 38898450 PMC11186201

[44] Tesfaye G, Loxton D, Chojenta C, Semahegn A, Smith R. Delayed initiation of antenatal care and associated factors in Ethiopia: a systematic review and meta-analysis. Reprod Health. 2017;14(1):150. doi: 10.1186/s12978-017-0412-4 29141675 PMC5688656

[45] Ahinkorah BO, Seidu A-A, Agbaglo E, Adu C, Budu E, Hagan JE Jr, et al. Determinants of antenatal care and skilled birth attendance services utilization among childbearing women in Guinea: evidence from the 2018 Guinea Demographic and Health Survey data. BMC Pregnancy Childbirth. 2021;21(1):2. doi: 10.1186/s12884-020-03489-4 33390164 PMC7778812

[46] Tolera H, Gebre-Egziabher T, Kloos H. Using Andersen’s behavioral model of health care utilization in a decentralized program to examine the use of antenatal care in rural western Ethiopia. PLoS One. 2020;15(1):e0228282. doi: 10.1371/journal.pone.0228282 31986187 PMC6984696

[47] Alemayehu M, Gebrehiwot TG, Medhanyie AA, Desta A, Alemu T, Abrha A, et al. Utilization and factors associated with antenatal, delivery and postnatal Care Services in Tigray Region, Ethiopia: a community-based cross-sectional study. BMC Pregnancy Childbirth. 2020;20(1):334. doi: 10.1186/s12884-020-03031-6 32487069 PMC7268454

[48] Haddrill R, Jones GL, Mitchell CA, Anumba DOC. Understanding delayed access to antenatal care: a qualitative interview study. BMC Pregnancy Childbirth. 2014;14:207. doi: 10.1186/1471-2393-14-207 24935100 PMC4072485

[49] Aziz Ali S, Aziz Ali S, Feroz A, Saleem S, Fatmai Z, Kadir MM. Factors affecting the utilization of antenatal care among married women of reproductive age in the rural Thatta, Pakistan: findings from a community-based case-control study. BMC Pregnancy Childbirth. 2020;20(1):355. doi: 10.1186/s12884-020-03009-4 32522258 PMC7288520

[50] Tang X, Ding L, Feng Y, Wang Y, Zhou C. Antenatal care use and its determinants among migrant women during the first delivery: a nation-wide cross-sectional study in China. BMC Pregnancy Childbirth. 2019;19(1):355. doi: 10.1186/s12884-019-2520-3 31615423 PMC6792185

[51] Hameed W, Asim M, Saleem S, Avan BI. Inequalities in utilisation of essential antenatal services for women with disabilities in Pakistan: analysis of a cross-sectional demographic and health survey of Pakistan 2017-2018. BMJ Open. 2023;13(7):1–11. 10.1136/bmjopen-2023-074262 PMC10373668 37487675

[52] Redshaw M, Malouf R, Gao H, Gray R. Disability in pregnancy womens experience. BMC Pregnancy Childbirth. 2013;13:174. 24034425 10.1186/1471-2393-13-174 PMC3848505

[53] Temane AM, Magagula FN, Nolte AGW. Midwives’ lived experiences of caring for women with mobility disabilities during pregnancy, labour and puerperium in Eswatini: a qualitative study. BMC Womens Health. 2024;24(1):207. doi: 10.1186/s12905-024-03032-z 38561691 PMC10986101

[54] Ganle JK, Otupiri E, Obeng B, Edusie AK, Ankomah A, Adanu R. Challenges Women with disability face in accessing and using maternal healthcare services in Ghana: a qualitative study. PLoS One. 2016;11(6):e0158361. doi: 10.1371/journal.pone.0158361 27347880 PMC4922658

[55] Devkota HR, Murray E, Kett M, Groce N. Are maternal healthcare services accessible to vulnerable group? A study among women with disabilities in rural Nepal. PLoS One. 2018;13(7):e0200370. doi: 10.1371/journal.pone.0200370 30005080 PMC6044538

[56] Wołowicz A, Kocejko M, Ferenc K. Women with disabilities and access to gynaecological services in Poland. Disabil Soc. 2022;37(3):386–405.

[57] Apolot RR, Ekirapa E, Waldman L, Morgan R, Aanyu C, Mutebi A, et al. Maternal and newborn health needs for women with walking disabilities; ‘the twists and turns’: A case study in Kibuku District Uganda. Int J Equity Health. 2019;18(1):1–10. 30866957 10.1186/s12939-019-0947-9 PMC6416885

